# Magnetic field-dependent shape anisotropy in small patterned films studied using rotating magnetoresistance

**DOI:** 10.1038/srep16139

**Published:** 2015-11-13

**Authors:** Xiaolong Fan, Hengan Zhou, Jinwei Rao, Xiaobing Zhao, Jing Zhao, Fengzhen Zhang, Desheng Xue

**Affiliations:** 1 The Key Lab for Magnetism and Magnetic Materials of Ministry of Education, Lanzhou University, Tianshui South Road 222, 730000 Lanzhou, People’s Republic of China

## Abstract

Based on the electric rotating magnetoresistance method, the shape anisotropy of a Co microstrip has been systematically investigated. We find that the shape anisotropy is dependent not only on the shape itself, but also on the magnetization distribution controlled by an applied magnetic field. Together with micro-magnetic simulations, we present a visualized picture of how non-uniform magnetization affects the values and polarities of the anisotropy constants 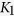 and 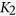. From the perspective of potential appliantions, our results are useful in designing and understanding the performance of micro- and nano-scale patterned ferromagnetic units and the related device properties.

Shape anisotropy, which originates from the dipole-dipole interactions that occur in ferromagnets, is a fundamental issue in magnetism^1^. For uniformly magnetized magnetic ellipsoids bodies, the shape anisotropy can be quantificationally described by the demagnetization tensor **N** which depends only on the shape. Thus, it provides a simple mechanism for designing the local effective anisotropy field of micro- and nano-scale magnetic units^2^,^3^,^4^. However, for an arbitrary magnet with a non-ellipsoid shape (such as the commonly used square and rectangle), it is difficult to realize uniform magnetization under a finite magnetic field^5^,^6^,^7^. As a consequence of nonuniform magnetization, the values of **N** that are estimated by uniformly magnetized ellipsoid approximation would be inaccurate and not suitable for describing shape anisotropy^8^,^9^. Moreover, a magnetic field-dependent shape anisotropy is expected. As more and more micro- and nano-scale patterned ferromagnetic units are used in spintronics devices, the shape anisotropy plays an increasingly important role in determining both the magnetic properties of local magnets and the related device performances^10^,^11^,^12^,^13^,^14^. Therefore, it is time to systematically investigate the shape anisotropy in micro- and nano-scale magnetic units, which is important in both physics and applications.

## Methods

The universal method for describing magnetic anisotropy is to determine the anisotropy constants 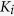 (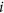 = 1, 2, …) in the expression of magnetic anisotropy energy 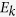. For uniaxial anisotropy, *E*_*k*_ = *K*_1_ 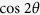 + K_2_ 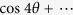, where 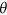 is the angle of the magnetization with respect to a certain symmetry axis. Many methods have been explored to measure magnetic anisotropy. Some methods can only give the first-order term 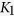 or the effective anisotropy field 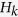 to estimate the anisotropy, such as the intersection method^15^, the area method^16^, and the singular point detection (SPD) method^17^,^18^ but these methods are not sufficiently accurate for our experiments. There are also a few methods that can give higher-order terms, such as the torque method^19^,^20^,^21^ and the magnetic resonance method^22^,^23^. It is worth noting that the ferromagnetic resonance (FMR) method is one of the best methods for determining magnetic anisotropy with very high sensitivity^24^,^25^. There are two kinds of FMR experiments, frequency-swept FMR and magnetic field-swept FMR. To obtain accurate anisotropy values, angular resolved measurements are needed, and 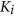 can be obtained by fitting the angle dependent resonance position curves (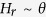 for field swept, 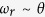 for frequency swept). Such method can be realized using traditional cavity FMR, transmission line or co-planar waveguide^26^,^27^,^28^,^29^,^30^,^31^,^32^, and DC electrical detection of FMR^33^,^34^,^35^,^36^,^37^,^38^,^39^,^40^,^41^,^42^,^43^,^44^, and has been widely used in various magnetic materials. Note that, the field-swept FMR method is accurate only when the anisotropy itself is independent of the applied magnetic field, such as the magnetocrystalline anisotropy. If 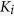 is a function of the applied magnetic field (i.e., the shape anisotropy in our case), such a method becomes “inaccurate” because the relation between 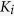 and 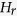 is now hidden in 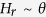 and that relation cannot be directly obtained from the fitting. In this work, by taking the advantage of frequency-swept FMR and the torque method that determining anisotropy at a certain magnetic field, and the advantage of direct electrical detection which is its inherent high sensitivity, we used a similar but experimentally simpler DC electrical method to quantify the magnetic field-dependent shape anisotropy in a small magnetic unit.

Two samples were used in the experiments. One was a 3 mm × 3 mm × 50 nm Co ferromagnetic film, and the other was a 3 mm × 50 *μ*m 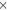 50 nm Co microstrip. Both samples were made of a Co film deposited on glass substrate using magnetron sputtering. The background pressure was lower than 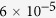 Pa and a pressure of 0.2 MPa of argon was used. The patterns were realized using laser exposure and the lift-off method. The magnetoresistance was measured by using lock-in techniques: a lock-in amplifier (SR830) provided a 2 V voltage with a modulation frequency of 1.31 kHz, and the sample was connected with a large resistance (20 kΩ) in series. At the same time, the lock-in amplifier measured the voltage 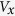 at both ends of the microstrip. Thus, the resistance of the microstrip can be calculated using the expression 
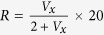
 k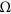. The magnetic hysteresis loops were measured using a vibrating sample magnetometer (VSM; EV9, MicroSense, Westwood, MA, USA).

## Results and Discussions

**Figure 1:**
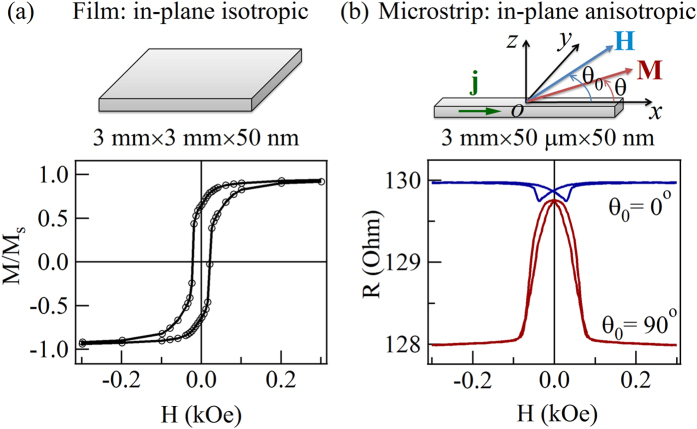
The magnetic properties of the Co film and Co strip. (**a**) The magnetic hysteresis loop of Co film. (**b**) The magnetoresistance curves of a Co micro-strip at different 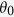.

First, we measured the magnetic hysteresis loops of Co film with in-plane applied magnetic field 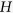 along different directions. All of the in-plane hysteresis loops were the same as shown in Fig. 1(a), which indicated that the Co film is in-plane isotropic. The saturation magnetization 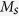 = 16.5 kOe and coercivity 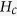 = 27.5 Oe. However, because of the very small volume of the microstrip (approximately 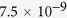 cm^3^), the maximal signal was approximately 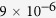 emu, which is close to the resolution of the VSM. Then we used anisotropy magnetoresistance (AMR) curves (magnetoresistance as a function of magnetic field 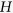) to identify the in-plane anisotropy of the microstrip, as shown in Fig. 1(b) where 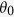 is the angle of the applied magnetic field with respect to the long axis of the Co micro-strip.The significant differences between the curves show that the Co micro-strip is magnetic anisotropic. Comparing the results of Fig. 1(a) with Fig. 1(b), we can conform that the anisotropy of the Co microstrip originated from the shape anisotropy, and that the effective anisotropy field is approximately 80 Oe. Then, the problem becomes the following: how do we get the accurate value of anisotropy field? As noted in the method section, the torque method can accurately measure anisotropy constants 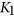 and 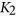 by fitting the torque curve. Can the rotating anisotropy magnetorsistance (RAMR) 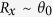 curves give the accurate value of the shape anisotropy? Before we introduce the experiment results, comprehension of the features of the RAMR curve is necessary.

In the following, we calculate the RAMR curve at different magnetic field amplitudes by using the model shown in Fig. 1(b). The expression of AMR is given by^11^


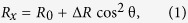


where, 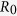 is ordinary resistance, 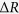 is the anisotropy magnetoresistance, and 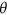 is the orientation of magnetization 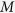 with respect to the current density 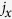. Our first task is to determine 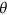 from the equilibrium condition when applying a magnetic field with amplitude 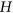 and direction 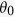. Based on the coherent rotation model, the total energy is given by


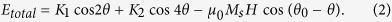


The equilibrium direction of magnetization is determined by the minimum of 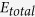 with respect to 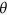, i.e. 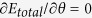 and 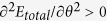,


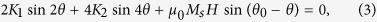



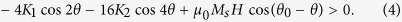


Considering 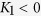 (i.e., the easy axis lies along the 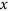 axis), the anisotropy field 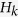 and reduced magnetic field 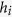 (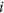 = 1, 2, 3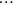) can be defined as:


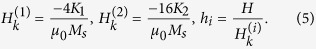


If we ignore 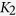 temporarily for a simple case, Eqs. (3) and (4) can be simplified as


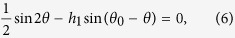



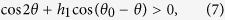


**Figure 2:**
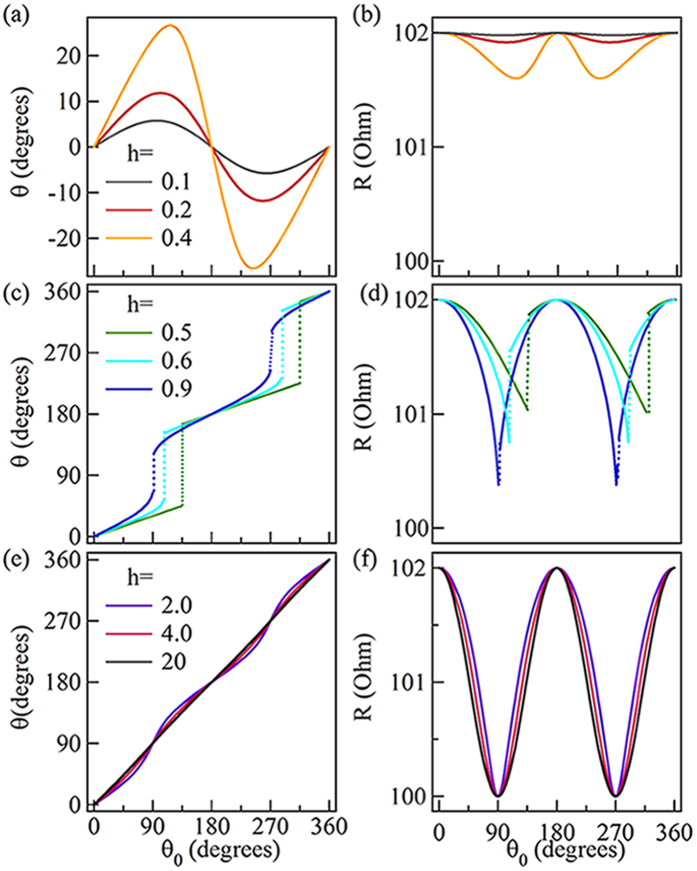
The calculated 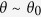 curves and RAMR curves. (**a**,**c**,**e**) The calculated 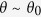 curves at different 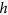. (**b**,**d**,**f**) The magnetoresistance (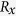) as a function of 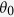 at different 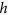.

Based on Eqs. (6) and (7), we can calculate the relation of 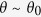 with any selected 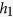 value. Putting 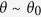 into Eq. (1), the angular dependence of the magnetoresistance (i.e., the RAMR curve 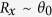) can be obtained. Supposing 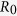 = 100  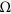, 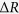 = 2  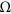, the 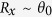 curves have been calculated at different 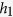; as shown in Fig. 2. Those curves can be divided into three groups with quite different features. Group 1: 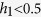, as shown in Fig. 2(a,b), both the 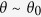 curves and 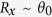 curves are continuous, and have a period of 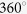, which indicates that the magnetization process is reversible under small applied magnetic fields. Group 2: 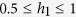; as shown in Fig. 2(c,d), the curves are obtained at relatively high applied fields. It is found that the periods of these curves are 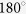 rather than 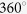, and that two “jumps” appear, which makes the curves discontinuous. The “jumps” results from an irreversible rotation of magnetization from current state to a more steady equilibrium state at critical values of 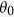
45. Group 3: 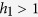; as plotted in Fig. 2(e,f), although the period is 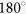, the curves become continuous again, which indicates that the magnetization is approaching the saturation state.

**Figure 3:**
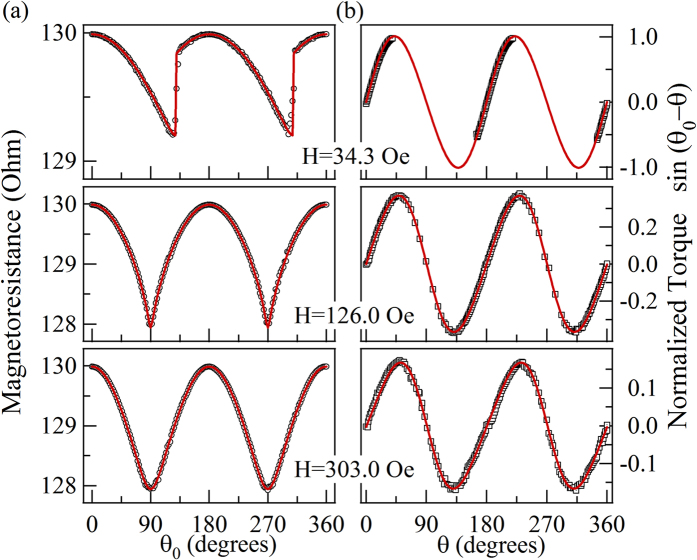
The RAMR curves and normalized torque curves obtained in different magnetic fields. (**a**) Symbols represent experimental 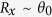 curves measured at 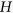 = 34.3 Oe, 126.0 Oe, and 303.0 Oe respectively; red lines are calculated curves used for corresponding fitted anisotropy constants. (**b**) The data of normalized torque curves 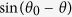 as a function of 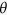; symbols are experimental data and the solid curves are fitting curves based on Eq. (8).

Immediately thereafter, we measured the RAMR curves of the Co micro-strip at different H, and the raw data (symbols) are shown in Fig. 3(a). The RAMR curve measured at *H* = 34.3 Oe has features similar to the second group’s theoretical curves shown in Fig. 2(d), wherein two obvious “jumps” can be observed. The curves measured at *H* = 126.0 Oe and 303.0 Oe look similar to those of the third group shown in Fig. 2(f). Therefore, our experimental results are basically consistent with the theory. We also measured the 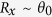 curve at *H* = 22 Oe, which should be corresponding to the case of the first group. However, as the applied magnetic field is relative small, the magnetization switching mechanism is domain wall motion rather than coherent rotation as shown in supplementary material. Thus, the cases 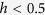 are not suitable for determining the anisotropy constants, we did not add the cases 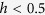 in this letter.

An accurate anisotropy field can be obtained by fitting the normalized torque curves 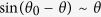, as shown in Fig. 3(b). The angle 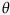 was calculated from the RAMR curves by using Eq. (1) with 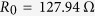 and 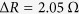, as determined previously. The fitting function is a transformation of Eq. (3), given by


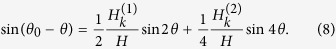


As shown in Fig. 3(b), the experimental results can be well fitted with that equation, which gives birth to the values of 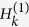 and 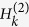. Moreover, the anisotropy constants 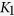 and 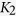 can be calculated using Eq. (5), as shown in Table 1. However, the values of 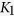 (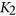) shown in the table differ from one another. To verify whether those values are correct, we took the values of 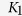, 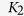 and 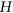 in the table back to Eq. (3) and (4), and then calculated the 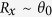 curves, i.e. the red curves shown in Fig. 3(a). The well matching between the experimental data and calculated curves indicates that all of the values shown in the table are correct. In the supplement, there is a discussion about the importance of higher-order terms in the fitting results, which is the circumstantial evidence of the accuracy of this method.

**Figure 4:**
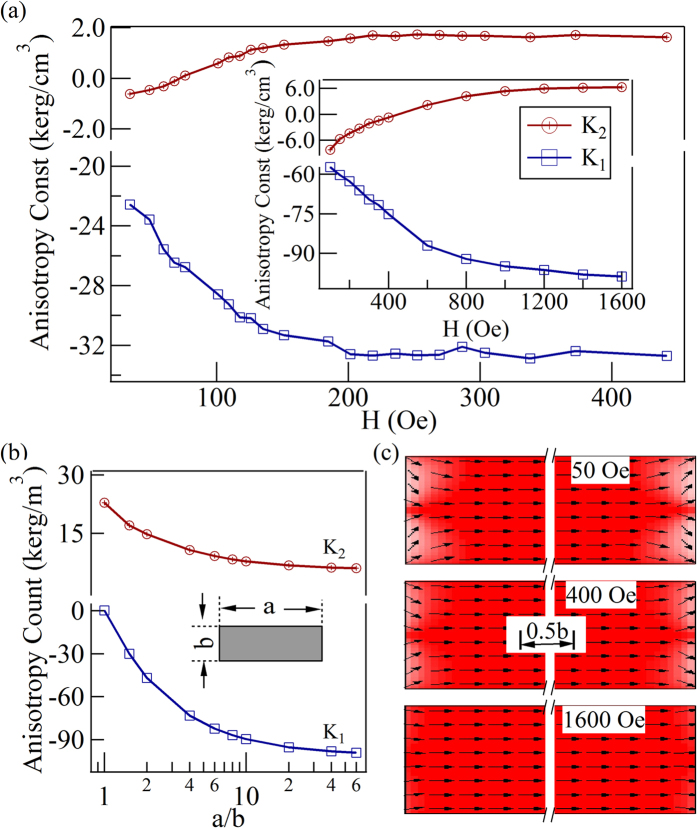
The magnetic field-and aspect ratio-dependent anisotropy constants. (**a**) The relationship between anisotropy constants and magnetic field; the insert is the simulative result. (**b**) The simulative 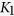 and 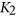 as a function of the rate of a:b. (c) The distribution of magnetic moments at different magnetic fields obtained by the micro-magnetic simulation.

The difference between 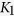 and 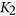 determined under different 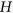 unveils a fundamental issue in magnetism: the shape anisotropy not only is determined by the shape itself, but also depends on the applied magnetic field. It has been written in every textbook of magnetism that the shape anisotropy of any non-ellipsoid magnet is a complicated issue because of the non-uniform magnetization. Therefore, it is not surprising that the shape anisotropy is field dependent. However, because the non-uniform magnetization cannot be quantified simply, theoretical calculations of shape anisotropy in non-ellipsoid magnets are quite complicated. Because accurate anisotropy constants can now be experimentally determined, we systematically measured the shape anisotropy constants at different 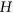 as shown in Fig. 4(a). It is found that when 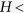 200 Oe, both the amplitudes of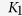 and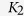 vary obviously with 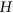; when H > 200 Oe, 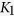 and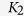 are saturated. Those experimental results can be further supported by the simulated 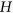 dependent 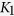 and 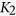, as in the insert shown in Fig. 4(a). We calculated the angular dependent demagnetization energy curves and fitted them with 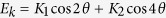 to determine the values of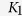 and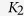. The OOMMF^46^ was used to simulate the demagnetization energy of a Co strip. In the simulations, the Co strip had a length 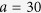 *μ*m, and width 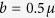
m, which has the same aspect ratio as our sample; the strip thickness was 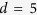 nm, and the unit cell size was 5 × 5 × 5  nm^3^. Material parameters used were typical for Co, namely, saturation magnetization 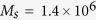 Am^−1^, and exchange stiffness constant 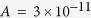 Jm^−1^ 47. As shown in Fig. 4(a), the simulative curves (inset) have the same tendency as the experimental curves, which indicates that the measured results of 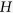-dependent 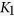 and 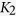 are reasonable.

To present a visualized picture of how the non-uniform magnetization affects the values and polarities of 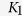 and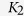, the distributions of 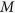 in a squareness ratio a/b = 60:1 Co strip were simulated with applied magnetic field 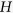 = 50 Oe, 400 Oe and 1600 Oe, as shown in Fig. 4(c). The arrows represent the direction of 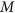, and the color stands for the amplitude of the magnetic moment along the strip. Obviously, the non-uniformed magnetization emerges at the edge of the strip. With an increasing magnetic field, the magnetization distribution becomes uniform, which can be directly linked to the 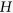-dependent 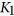 and 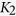. Therefore, for non-uniformly magnetized magnets, the shape anisotropy depends not only on the shape, but also on the magnetization distribution controlled by the applied magnetic field. In the following, we want to discuss the polarity of the anisotropy constants. Both the experimental and simulation results show that 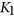 and 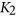 have opposite polarity (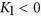 and 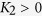) in the saturation status. The polarity actually represents the direction of the easy axis. To illustrate this argument, we simulated the values of 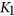 and 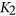 as functions of the squareness ratio with a sufficiently large applied magnetic field 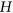 = 1600 Oe as shown in Fig. 4(b). When the squareness ratio is 1:1, 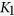 = 0, 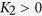, the anisotropy energy can be written as 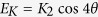, and the minimum of 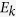 appears at 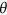 = 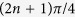 i.e. the easy axis is along the cater-corner, which is consistent with the previously reported results^47^. When the squareness ratio 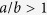, 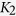 decreases slightly and 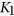 appears with negative values. The amplitude of 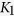 increases rapidly and becomes dominant, which means that the easy axis is along the strip.

**Figure 5:**
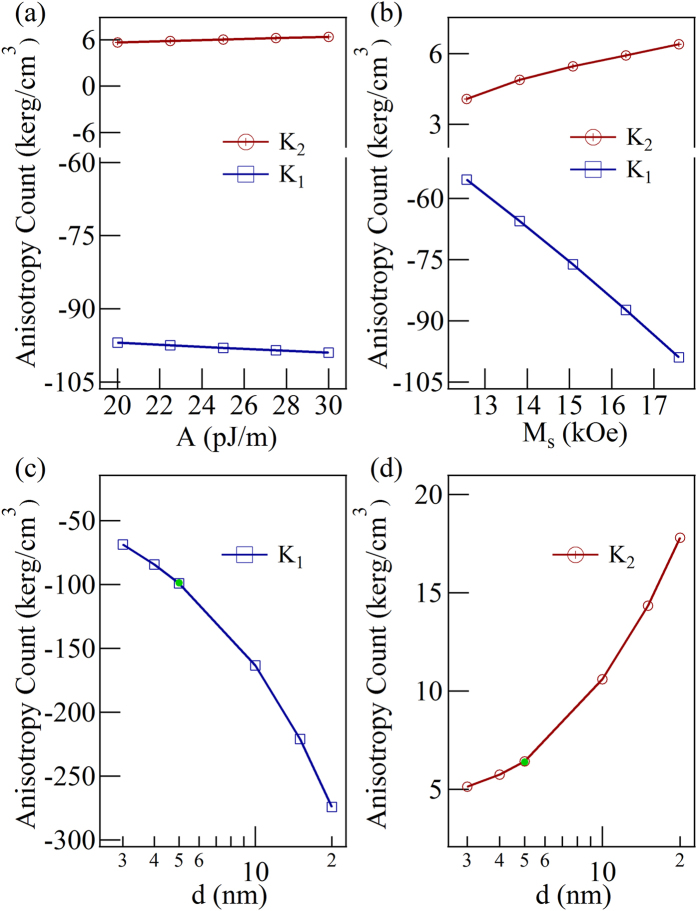
The simulated anisotropy constants change with the exchange stiffness constant, saturation magnetization and thickness. (**a**) The relationship between anisotropy constants and the amplitude of the exchange stiffness constant. (**b**) The simulated 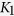 and 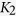 as a function of the saturation magnetization. (**c**) The thickness dependent anisotropy constant 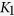. (**d**) The thickness dependent anisotropy constant *K*_2._

We studied the influences of effective exchange stiffness constant 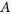, saturation magnetization 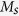, and film thickness 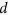 on the values of the shape anisotropy obtained at saturation magnetized condition using simulation. We changed one parameter for the simulation while the other parameters remained the same as the previous ones. As show in Fig. 5(a), the anisotropy constants did not vary significantly while 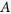 changed from 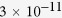 Jm^−1^ to 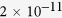 Jm^−1^. As show in Fig. 5(b), while the 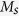 changed from 12.6 kOe (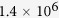 Am^−1^) to 17.6 kOe (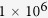 Am^−1^), the amplitude of 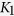 and 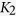 increased almost linearly with 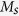, which is the nature of demagnetization energy. Moreover, the simulations with the cases where 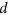 = 3 nm (cell sizes = 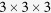 nm^3^), 4 nm (cell sizes = 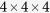 nm^3^), 5 nm, 10 nm, 15 nm, and 20 nm (cell sizes = 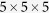 nm^3^) were performed, as shown in Fig. 5(c,d). Additionally, the green points represented the simulated results of 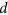 = 5 nm using smaller cell size 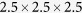 nm^3^, which is consistent with previous case. Therefore, the influence of the unit cell size on the simulation results can be ignored. It should be noted that the simulated values for 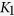 (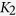) in Fig. 4(a) are different from the experimental ones, the main reason comes from the thickness we used for the simulations. The size of the experimental sample was 3 mm 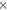 50 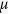
m × 50 nm; to meet the proportion, the size of the simulated strip should be 30 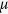
m × 0.5 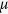
m × 0.5 nm. However, if we use cell sizes = 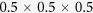 nm^3^, the simulation would be far beyond our calculation ability. According to the tendency shown in Fig. 5(c,d), the value of 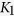 (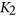) may reach −35 kerg/cm^3^ (2.2 kerg/cm^3^) when 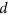 = 0.5 nm, which is close to the experimental values.

In fact, the shape anisotropy, which originates from the dipolar-dipolar interaction, is an old but fundamental problem in magnetism. The difficulty in calculating of shape anisotropy in non-ellipsoid magnets comes from the fact that non-uniform magnetization cannot be simply quantified. Because we can now quantify accurately the shape anisotropy which is closely related to the non-uniform magnetization, the magnetic field-dependent 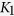 and 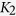 determined by our method may be semi-quantificational parameters for describing non-uniform magnetization. From the application perspective, both shape anisotropy and non-uniform magnetization are important in designing and understanding the performance of micro- and nano-scale patterned ferromagnetic units as well as the related device properties.

## Conclusions

In conclusion, based on the RAMR method, the shape anisotropy of a Co micro-strip was systematically studied. It was found that using only demagnetization factor is not sufficient to describe the shape anisotropy of non-ellipsoid magnets, and that non-uniform magnetization would result in an 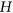-dependent anisotropy. These results have also been demonstrated through micromagnetic simulations.

## Additional Information

**How to cite this article**: Fan, X. *et al.* Magnetic field-dependent shape anisotropy in small patterned films studied using rotating magnetoresistance. *Sci. Rep.* **5**, 16139; doi: 10.1038/srep16139 (2015).

## Supplementary Material

Supplementary Information

## Figures and Tables

**Table 1:** The magnetic anisotropy parameters measured at different *H*.

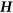	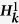	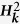	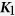	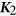
(Oe)	(Oe)	(Oe)	(kerg/cm^3^)	(kerg/cm^3^)
34.3	68.8	7.3	−22.58	−0.60
126.0	92.0	−13.6	−30.20	1.12
303.0	99.0	−20.8	−32.50	1.71
