# Supplementary material for: Magnetic field-dependent shape anisotropy in small patterned films studied using rotating magnetoresistance
Source: Sci Rep. 2015 Nov 13;5:16139. doi: 10.1038/srep16139 (PMC4643335; doi:10.1038/srep16139)
Supplement: Supplementary Information [file srep16139-s1.pdf]

Magnetic field-dependent shape anisotropy in small patterned films studied using rotating magnetoresistance

Author: Xiaolong Fan*, Hengan Zhou, Jinwei Rao, Xiaobing Zhao, Jing Zhao, Fengzhen Zhang, and Desheng Xue
The Key Lab for Magnetism and Magnetic Materials of Ministry of Education,
Lanzhou University, Lanzhou 730000, People's Republic of China, a)email: fanxiaolong@lzu.edu.cn

Supplemental Material

S1、Two different expressions of anisotropy energy.

There are two different Fourier expansions of anisotropy energy E_k as $E_k = K_1 \cos 2\theta + K_2 \cos 4\theta + \cdots$ and $E'_k = K'_1 \sin^2\theta + K'_2 \sin^4\theta + \cdots$. They are the same in essence in determining the equilibrium state of M_s:

$$\begin{aligned} E_k &= K_1 \cos 2\theta + K_2 \cos 4\theta \\ &= K_1(1-2\sin^2\theta) + K_2[2(1-2\sin^2\theta)^2 - 1] \\ &= (-2K_1 - 8K_2)\sin^2\theta + 8K_2 \sin^4\theta + (K_1 + K_2) \\ &= K'_1 \sin^2\theta + K'_2 \sin^4\theta + \text{Cont.} \end{aligned} \quad \text{Sq(1)}$$

i.e., $K'_1 = -2K_1 - 8K_2$, $K'_2 = 8K_2$.

If use $E'_k = K'_1 \sin^2\theta + K'_2 \sin^4\theta + \cdots$, the expression of total energy i.e. Eq(2) in the manuscript should be changed as:

$$E'_{total} = K'_1 \sin^2\theta + K'_2 \sin^4\theta - \mu_0 M_s H \cos(\theta_0 - \theta) \quad \text{Sq(2)}$$

The minimum of E'_{total} is determined by

$$K'_1 \sin 2\theta + 4K'_2 \sin^3\theta \cos\theta - \mu_0 M_s H \sin(\theta_0 - \theta) = 0 \quad \text{Sq(3)}$$

$$2K'_1 \cos 2\theta + K'_2\left(3\sin^2 2\theta - 4\sin^4\theta\right) + \mu_0 M_s H \cos(\theta_0 - \theta) > 0 \quad \text{Sq(4)}$$

Defined the anisotropy field $H_{k'}$ and $h_{i'}$ as

$$H_{k'}^{(1)} = \frac{2K'_1}{\mu_0 M_s}, H_{k'}^{(2)} = \frac{4K'_2}{\mu_0 M_s}, \ h_{i'} = \frac{H}{H_{k'}^{(i)}} \quad \text{Sq(5)}$$

Temporarily ignoring K'_2 for simplifying the calculations, Sq(3) and Sq(4) can be simplified as

$$\frac{1}{2}\sin 2\theta - h_{1'} \sin(\theta_0 - \theta) = 0 \quad \text{Sq(6)}$$

$$\cos 2\theta + h_{1'} \sin(\theta_0 - \theta) > 0 \quad \text{Sq(7)}$$

The accurate anisotropy fields can also be obtained by fitting the normalized torque curves as shown in Fig.3(b) in the manuscript with

$$\sin(\theta_0 - \theta) = \frac{1}{2}\frac{H_{k'}^{(1)}}{H}\sin 2\theta + \frac{H_{k'}^{(2)}}{H}\sin^3\theta\cos\theta \quad \text{Sq(8)}$$

However, the expression $E_k = K_1 \cos 2\theta + K_2 \cos 4\theta + \cdots$ has unique advantages in presenting the magnetic symmetry. $K_1 = 0$ and $K_2 \neq 0$, as an example, E_k presents a pure cubic anisotropy. On the other hand, if $K'_1 = 0$ and $K'_2 \neq 0$, for $E'_k = K'_2 \sin^2\theta + K'_2 \sin^4\theta + \cdots$, it contains not only an cubic anisotropy, but also an uniaxial anisotropy.

S2、The influence of the second anisotropy constant K_2 on the fitting results.

Although, the value of K_2 is usually smaller than K_1, taking the K_2 in accurately determining anisotropy is necessary. As shown in Fig.S1, the symbols are experimental RAMR curves measured at H =34.3 Oe,

126.0 Oe, 303 Oe, the solid lines are corresponding calculated RAMR curves based on the fitting parameters. Here, the red curves are the cases considered both K_1 and K_2, and the blue curves only consider K_1. It is clear to us that the red curves fit more well than the blue curves do *i.e.* K_2 do play an important role in the magnetic anisotropy and should not be ignored in our case.

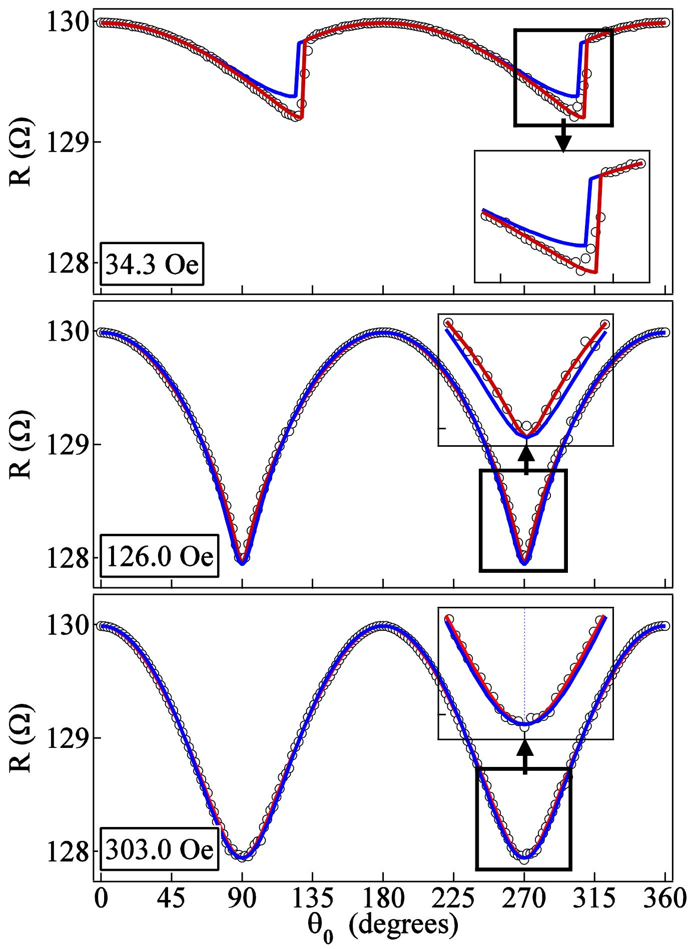

Fig.S1: The symbols are experimental RAMR curves measured at H =34.3 Oe, 126.0 Oe, 303 Oe, the solid lines are corresponding calculated RAMR curves.

S3、The case of *h*<0.5

In the experiment, we measured the $R_x \sim \theta_0$ curve at H= 22 Oe as shown in the supplementary material (see Fig. S2), which should be corresponding to the case $h < 0.5$. However, we can find that the curve is not the same as the calculated curve $h = 0.4$. This is because that when the applied magnetic field is relative small, the magnetization switching mechanism is tend to be the domain wall motion rather than coherent rotation. The Stoner-Wohlfarth model is not suitable for our sample in the case $h < 0.5$. In order to verify the magnetization switching mechanism, the magnetoresistance curves at different angles were measured; each curve has a dip, as shown in Fig. S3(a). Such a dip is attributed to magnetization switching in the microstrip. H_S is the switching field. The angular dependence of the normalized switching field, $\mathrm{H_S}(\theta_0)/\mathrm{H_S}(\theta_0 = 0)$ is shown in Fig. S3(b). This latter figure demonstrates that this ratio gradually increases with angle θ_0, a result that can be understood on the basis of the Kondosky mode. In this model, switching is attributed to domain wall motion. [APL 95, 062511 (2009)]

Based on above data, the rotating magnetoresistance method would not be functional for the case h<0.5, therefore, we did not add the case of h<0.5 in the manuscript.

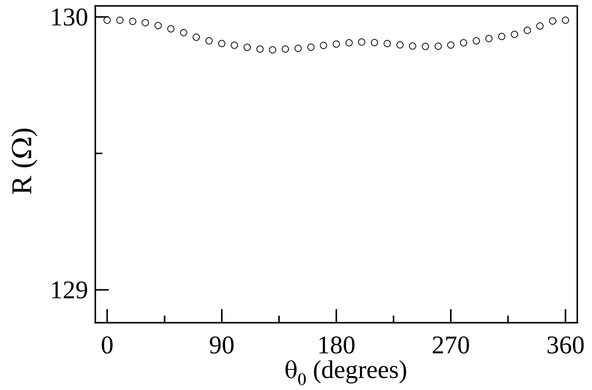

Figure S2: The R_x~θ_0 curve measured at H= 22 Oe.

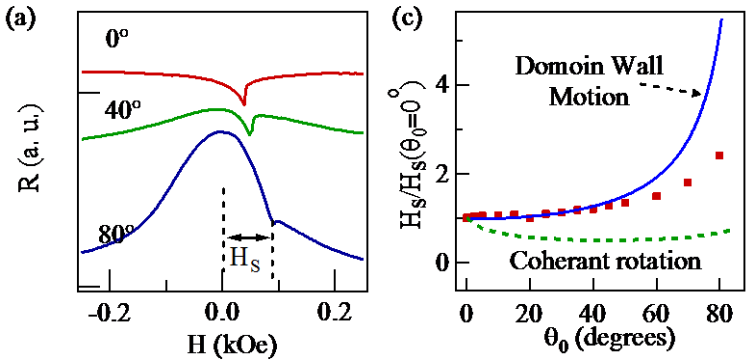

Figure S3: (a)The magnetoresistance curves measured at $\theta_0 = 0^o$、40°、80°; (b)Angular dependence of normalized switching field: Experimental data (symbols), and theoretical results based on the Kondosky model (solid line) and Stoner–Wohlfarth model (dashed line).
